# Empowering Ukrainian healthcare and humanitarian aid workers: cocreating a peer support staff wellbeing curriculum

**DOI:** 10.3389/fpubh.2025.1654263

**Published:** 2025-10-01

**Authors:** Courtney Welton-Mitchell, Nawaraj Upadhaya, Andrew Riley, Julius Torres Kellinghusen, Alexa Hansen, Halyna Skipalska, Peter Navario, Theresa P. Castillo

**Affiliations:** ^1^Department of Environmental and Occupational Health, Colorado School of Public Health, CU Anschutz Medical Campus, Aurora, CO, United States; ^2^HealthRight International, New York, NY, United States; ^3^Center for Operational Analysis and Research (COAR), Nicosia, Cyprus; ^4^HealthRight International, Kiev, Ukraine

**Keywords:** wellbeing, healthcare, humanitarian, Ukraine, peer support, curriculum, survey, participatory

## Abstract

In February 2022 Russia launched an invasion of Ukraine. National aid workers responded to the crisis, at the risk of their own wellbeing. This case study details the cocreation of a peer support intervention by a global public health non-profit working with national staff in Ukraine. As a first step in peer support wellbeing curriculum development, an online survey was developed and administered for 530 Ukrainian healthcare and humanitarian aid workers. The survey resulted in 300 valid responses, for a 57% response rate. Top stressors included: *personal safety and security* (43%), *concerns for family and friends* (32%), and *financial hardships* (29%). Just over one-third of respondents indicated that stress was interfering with their ability to do their job. Common forms of coping included *distraction* (73%), and *use of alcohol or drugs* (32%). Nearly all (97%) indicated interest in a peer support group intervention. In addition to interest in socializing with colleagues, top preferences for curriculum content included: *coping skills, psychological preparedness, peer support facilitation skills, and healthy team dynamics*. Survey results were used to develop a 6-module peer support group curriculum, refined through subsequent workshops. This participatory approach can be utilized to develop tailored wellbeing curriculum for workers of various types and across settings.

## Highlights

- Focused on Ukrainian healthcare and humanitarian aid workers, this case study provides a roadmap for cocreating a tailored peer support curriculum.
- Workers in Ukraine emphasized intervention preferences through a survey and follow on workshop that significantly shaped the peer support intervention.
- While this example is specific to national workers with a global non-profit in Ukraine, a similar process can be followed for workers of various types and across settings.

## Introduction

### Context

In February 2022 Russia launched an invasion of Ukraine. Through September 15, 2025, over 40,000 civilian casualties have been verified by the Office of the High Commissioner for Human Rights (OHCHR), as a result of over 3 years of ongoing invasion (1, 2). Additionally, there are currently 3.7 million internally displaced persons in Ukraine, and another 6.9 million refugees outside of the country (3). The conflict has had a negative impact on mental health throughout Ukraine, resulting in increased suicidal thoughts, feelings of helplessness, depression, and anxiety among the general population (4, 5). Healthcare and other humanitarian aid workers are suffering from increased rates of post-traumatic stress disorder, depression, anxiety, and other mental health concerns (6). Staff wellbeing interventions are urgently needed to care for the carers and enable them to address the needs of others. This article details an approach to cocreation of a peer support group intervention that may provide a useful roadmap for others to follow.

The healthcare and human rights organization supporting the work that is the focus of this article has been providing services in Ukraine since 2005. This includes housing, health care, and social support to over 400,000 people affected by violence (7). “Ukraine War Relief” services provided by staff with the organization include 80+ mobile crisis teams throughout the country. Through such teams staff provide mental health and psychosocial services including crisis counseling, legal aid, gender-based violence assistance, and child protection—including protection from trafficking and other forms of abuse. General medical support is also provided—including HIV care and support for pregnant women and new mothers evacuated from areas of active conflict. Additionally, essential supplies are provided including blankets, hygiene products, and non-perishable food items.

Under the “Caring for the Carers” (C4C) umbrella (an organization-wide staff wellbeing initiative), the organization also supports national healthcare and humanitarian aid workers through counseling, employee wellbeing policies, and safe space programming. With a growing workforce in Ukraine (over 600 staff), many directly and indirectly exposed to the ongoing violence, the organization has continued and increased staff wellbeing initiatives in recent years. This community case study details the cocreation of a staff wellbeing intervention during 2022–2024 by a global public health nonprofit working in Ukraine during a period of escalating violence in the Russo-Ukrainian War.

### Background

Healthcare workers and other humanitarian staff working in conflict settings have substantially higher risk of adverse mental health outcomes than many other professionals (8, 9). Several studies have documented high rates of depression, anxiety, post-traumatic stress disorder (PTSD), substance abuse and feelings of helplessness among such professionals (9–12). The risk may be even higher for national and local aid workers struggling with personal impacts of conflicts and disasters, faced with the suffering of one's own community members, and often lacking adequate resources to meet identified needs (12).

The risk of poor mental health for aid workers is driven largely by stressors facing such professionals in complex humanitarian crises. Research suggests common stressors include both chronic and acute challenges such as witnessing the suffering of others, working in insecure environments and experiencing personal threats, concerns over job security, and financial stress (11, 13–15). As a result, aid workers in crisis settings are at increased risk of burnout and are more likely to resign or change careers compared to those in other professions (12, 16). Such job-stress-related attrition can be costly for employers, considering the need to invest in training new staff (17). Those who stay on the job while not at their best may underperform, or can even endanger others (18). Such outcomes are not inevitable, however, even in high-risk job sectors. Employers can be proactive, mitigating risks with prevention strategies designed to bolster employee wellbeing and promote resilience (19).

Wellbeing at work has been an increasing area of emphasis in recent years. For example, one of the 2030 UN Sustainable Development Goals (goal 3) focuses on wellbeing, including at work. This is in line with biopsychosocial models of mental health emphasizing the influence of work factors on wellbeing (20–22). Additionally, work-specific burnout frameworks, such as the Job Demands-Resources (JD-R) model, illustrate how work factors (high demands, low resources) can result in poor mental health in the form of employee exhaustion and disengagement (23). Such theoretical models can assist in framing challenges and developing solutions to enable workers to thrive, including those in high risk professions.

Although not specific to armed conflict settings, such as the current case study with workers in Ukraine, various workplace wellbeing standards, frameworks and tools are also useful for developing interventions designed to mitigate workplace stress. For example, the National Standards of Canada for Psychological Health and Safety in the Workplace is designed to prevent psychological harm at work, with recommended programs, policies, benefits, training, and assessments (24). In the United States, the National Institute for Occupational Safety and Health (NIOSH) promotes a Total Worker Health Program, emphasizing injury and illness-prevention efforts to advance worker wellbeing, including a focus on mental health (25, 26). Additionally, the National Academy of Medicine has developed a National Plan for Healthy Workforce Wellbeing designed to reduce trends in health worker burnout (27). Globally, the United Nations has developed a wellbeing strategy that emphasizes the importance of staff access to mental health services (28). Additionally, the World Health Organization has been a leader in emphasizing the importance of protecting workers from risks, including psychological harm, and prioritizing specialized occupational health services (29). The Antares Foundation's “Managing Stress in Humanitarian Workers, Guidelines for Good Practice,” includes an 8-stage roadmap for mitigating distress and encouraging wellbeing among humanitarian aid workers (30). Taken together, such global frameworks can provide useful guidance for employers wanting to support their workforce, including healthcare and other humanitarian aid workers responding to complex crises.

Preventative strategies to support worker wellbeing, mitigate occupational stress, reduce the risk of burnout, and enhance work life balance, can be more effective than response-only approaches (19). Comprehensive prevention approaches can include injury prevention measures, exercise and diet programs, alcohol and drug prevention campaigns, healthy coping skills promotions, and supportive peer, family and community relationship building initiatives (31, 32). Prevention includes proactive approaches to designing interventions that can build capacity within groups of workers to support one another. Encouraging robust peer support networks is one way of going about this.

Relationship building among workers can happen through collaborative support frameworks. Peer-based support models can emphasize healthy coping skills while simultaneously promoting community cohesion and social support. Social support among peers is one of the most robust predictors of positive mental health outcomes, and is often a key component of effective interventions (33, 34). Peer support has been associated with wellbeing and decreased likelihood of burnout among various professionals, including frontline humanitarian aid workers (8, 35–37). Peer support interventions have also been used successfully with mental health professionals to address secondary stress and vicarious trauma (38). In one study researchers in Sierra Leone trained ex-Ebola Treatment Center staff to provide CBT-based group interventions to their peers to mitigate the risk to mental health of working in the high-stress environment. Results indicated improvement across all mental health outcomes, suggesting peer-led group interventions can be effective in reducing distress (39). Although peer support can be provided one on one, models using a group format may be especially beneficial, harnessing the power of the group. Group-based models have been shown to be effective for stress reduction and in treating symptoms of depression, anxiety, and PTSD, including in the workplace, and specific to burnout (40–45).

Peer support and participatory approaches to intervention design are well aligned. Codesigning interventions with those the intervention is intended to benefit may also increase the likelihood of intervention “fit” (acceptability, feasibility). Participatory approaches have been flagged as a key element in the development of interventions that encourage knowledge acquisition and transfer (46). Design of workplace wellbeing interventions through worker participation have the potential to empower employees, resulting in more sustainable approaches with content better suited to specific settings (47). Such participatory cocreation of interventions, coupled with peer support, is in line with recommended survivor and community-led response (SCLR) guiding principles. In SCLR those with the most to gain or lose are encouraged to take the lead in identifying what is needed and delivering services (48).

### Current study

The worker wellbeing approach described in this manuscript adheres to best practice guidelines and is in line with promising approaches to peer and group-based models. It moves beyond individual therapy frameworks focused on expatriate staff, to foreground national and local staff working on the frontlines of humanitarian response during a period of ongoing violence in the Russo-Ukrainian War. It addresses a need to cocreate a preventive peer-lead wellbeing intervention for staff exposed to traumatic events in emergency settings. Furthermore, it addresses a broader gap in the literature in describing how to cocreate and tailor peer support group interventions for a variety of workers and contexts.

## Methods

An online survey was developed and administered for 530 Ukrainian healthcare and other humanitarian aid workers (including many psychologists and social workers), working primarily in mobile outreach to those impacted by the Russo-Ukrainian War. The primary purpose of the survey was to gauge needs and determine interest in a staff wellbeing peer-based group intervention, to be developed based on staff input. The survey focused on distress; stressors; coping; social support; burnout and work satisfaction; benefit-finding and post-traumatic growth; organizational support; service utilization; and suggestions for a group-based peer support intervention.

### Materials and measures

An anonymous survey was designed to solicit feedback from Ukrainian healthcare and humanitarian aid workers. The survey included sections on *demographics; distress; stressors; coping; social support; burnout and work satisfaction; benefit-finding and post-traumatic growth; organizational support; service utilization; and suggestions for a group-based peer support intervention*. Although some survey content was adapted from existing measures, other content was investigator-created for the purpose of informing intervention development, in line with similar approaches used for staff wellbeing surveys. Investigator-developed items included questions on mental health history, threat to life, and sources of stress, drawing on previous staff wellbeing work by the lead author (49). A single item stress measure was utilized (*Please describe how much stress you have been experiencing in the last 2 weeks*, 1–10 scale), similar to items that have been shown to be valid for a variety of purposes (50). Coping items were adapted from the Brief COPE and included five items focused on actively seeking solutions, distraction, acceptance, denial, and use of alcohol or drugs (51). Social support items were adapted from the Social Support Scale (SSC) and included three items focused on emotional and instrumental support (such as help with daily tasks) (52). Burnout/Satisfaction from Work items were adapted from the ProQOL-5, with a focus primarily on compassion satisfaction and burnout (a subset of compassion fatigue), utilizing six items emphasizing satisfaction, feeling able to make a difference, work/life balance, feelings of being worn out and overwhelmed, feeling depressed because of suffering of others, feeling trapped by my job (53). Benefit-finding/Post-traumatic Growth items were adapted from the Post-traumatic Growth Inventory (PTGI), utilizing 6 items, with an emphasis on relating to others, new possibilities, personal strength, and appreciation of life (54). *See* ***Results*** *section for additional details related to measures*.

### Survey tool translation

The lead author and third author worked with staff in Ukraine to ensure that the survey included culturally and contextually appropriate phrasing and content. It was initially discussed at length with Ukrainian colleagues working with the global non-profit. Subsequently, it was translated, back translated, and further refined through an iterative process in collaboration with Ukrainian staff. This included online sessions to adapt the survey before finalizing. Once deployed, staff were given a Ukrainian language version of the survey, although they could choose to take the English version of the survey if preferred by selecting an option in the survey software toolbar.

### Procedure: survey administration

The Qualtrics survey link was sent by organizational leadership to all staff in Ukraine in late October 2022 and remained open for responses until late November 2022. Reminders were sent to staff twice asking them to complete the voluntary survey during the 1-month period. The survey included an initial section explaining the purpose—to better understand staff wellbeing needs and to inform services to meet identified needs. A definition was provided for “staff wellbeing,” focused on the overall quality and experience at work, including psychological, social, physical, material, and financial components. The survey was estimated to take about 20 min to complete.

### Ethical considerations

Survey front matter emphasized confidentiality and consent, including the anonymous and voluntary nature of the survey, with the data collected at only one measurement point. Participants were told they could skip items or stop at any time with no adverse consequences. The survey was used for the sole purpose of developing a staff wellbeing intervention, not to test or evaluate the intervention. No identifiable information was collected from participants, and the survey did not otherwise exceed typical “minimal risk” criteria. Data was stored with no identifiers, including removal of IP addresses. Access limitations and encryption measured were used even for the deidentified data.

After the initial data collection, the non-profit decided to publish an article focused on the peer support design process (as a case study). An IRB review was not required for the original purpose. In the process of preparing the manuscript, however, the authors utilized a university-based ethical review board tool to further determine what may be needed in this situation. IRB QI/Program Evaluation Self-Certification Tool was used to determine if this project falls outside the scope of what requires IRB review. The results related to this specific project conclude with the following statement: “*This project appears to constitute QI (quality improvement) and/or program evaluation and IRB review is not required because, in accordance with federal regulations, your project does not constitute research as defined under 45 CFR 46.102(d). If the project results are disseminated they should be characterized as QI and/or Program Evaluation findings*.”

### Analysis

Prior to analysis the data was cleaned. Incomplete responses, under the 50% completion threshold, were removed (36 cases), resulting in 300 viable surveys. Some sections included composite analyses across a few items (sum or average). In such cases those with missing data in these sections were excluded from analyses.

## Results

### Demographics

Of the 530 surveys disseminated, 300 valid responses were received, for a 57% response rate. The gender distribution of participants was as follows: women 75%, men 21%, other 1%, prefer not to answer 3%. All respondents were Ukrainian. Nearly all survey respondents were based in Ukraine 99%, with 1% working outside of Ukraine at the time of the survey. The breakdown by job sector was: Program = 90%; Operations = 7%; M&E/Research = 1%; Other = 2%. The 90% of program staff included significant numbers of psychologists and social workers engaged in programming related to mental health and psychosocial support services, gender-based violence and child protection. Additional program staff included doctors, nurses, and lawyers. Nearly half of staff completing the survey held program officer or assistant program staff positions (42%), with a small percentage in managerial positions (13%). In total, 64% were full-time, 19% were part-time and 17% were working as consultants. Most staff (up to 87%), were primarily engaged in in-person direct community interactions in the typical course of performing their work. Although the majority of staff were working in-person, in an office or community setting, 8% were fully remote.

### Mental health and stressors

#### History of mental health concerns

Respondents were asked, “have you been struggling with mental health challenges at any time before February 2022? (anxiety, depression, post-traumatic stress disorder, substance abuse, or other concerns) If yes, feel free to elaborate.” Of the total survey respondents, 11% (*n* = 32) indicated a history of mental health concerns *before Feb 2022*. When asked to elaborate, anxiety, depression and PTSD were emphasized in many responses, with anxiety listed most frequently. A few shared that fear related to COVID-19, concerns for the future, job stress, family conflicts, and death of loved ones were drivers of past mental health challenges.

#### Global distress

When asked to “describe how much stress you have been experiencing in the last 2 weeks, where 1 = I'm feeling calm, 5 = I'm feeling somewhat stressed, and 10 = I'm completely stressed out/overwhelmed,” the mean response was 3.77 with a median of 4 and a mode (most frequent number) of 5. A total of 45% of survey respondents are “somewhat stressed” or greater (referring to those who selected 5 or above; 32% selected 5).

#### Threat to life

When asked, “in the course of doing your job—have you ever felt that your life was in danger?” 24% indicated Maybe and 9% indicated Yes (*n* = 72, 28). When asked, “have you witnessed others killed, tortured or seriously injured?” 4% said Maybe, and 3% said Yes (*n* = 11, 9).

#### Greatest sources of stress

When asked to, think about your greatest source of stress currently. “Is this stress resulting from your job/job-related?” 22% indicated maybe and 8% indicated yes (*n* = 65, 23). An additional 16% indicated “I don't know” (*n* = 48).

#### Stress interfering with job

When asked, “is this stress interfering with your ability to do your job?” 22% indicated maybe and 7% indicated yes (*n* = 66, 22). An additional 7% indicated “I don't know” (*n* = 21).

#### Sources of stress

Survey respondents were asked, “are any of the following a source of stress for you?” Sources of Stress included 12 options, including an “other, specify” category. Top stressors rated by participants included: personal safety and security (a concern for 43%), concern for family and friends (32%), and financial hardships (29%), see Table 1. Additional qualitative responses for the “other, specify” category included stressors that may be related to the conflict: *Power outages, air alarms/sirens, being surrounded by death, war in Ukraine, death of a loved one, uncertainty about the future, and family problems*. Specific work-related qualitative responses included: *difficulty maintaining boundaries between work and nonworking hours, feeling guilty about not being able to provide enough to others given the needs of community members, including “mental health needs are great*.”

**Table 1:** Sources of stress.

**Item: *are the following a source of stress for you?* (*N* = 300)**	***N***	**%**
1. Safety and security concerns (*outside of work*)	125	43
2. Concerns about family and/or friends	93	32
3. Financial concerns	84	29
4. Safety and security concerns (during work)	81	28
5. Health concerns	77	26
6. Workload	55	19
7. An inability to contribute to work-related decisions/decision-making	41	14
8. Working hours	40	14
9. Mental health concerns	34	12
10. Other sources of stress	34	12
11. An inability to achieve work goals and objectives	23	8
12. Work-related conflicts (with supervisors, supervisees, and/or colleagues)	22	8

#### Coping and social support

The survey included questions about typical ways that respondents were coping with stress. The most frequent form of coping was Distraction (73% indicated they use this to cope with stress some or most of the time); Active coping/seeking solutions (68% indicated use of this approach some or most of the time); Pretending difficult things didn't happen (38% use some or most of the time, with another 30% using this a little of the time); Using alcohol or drugs to cope (32% indicated using this a little or some of the time). Most staff reported robust social support in their work and/or personal lives, indicating they have received at least some emotional support when needed (96%); they have someone to listen when they need to talk (81% most/all of the time); and they have someone to count on for support with daily tasks (77% most/all of the time).

#### Burnout/work satisfaction

Survey items focused on agreement with a series of statements (“in terms of your work, please indicate how much you agree with the following…”), including: satisfaction from helping, belief in making a difference through work, able to balance work and personal life, feeling worn out and overwhelmed, feeling depressed because of suffering of others, feeling trapped by work. The 5-point scale for these items is from “never” to “very often.” 1 = never, 2 = rarely, 3 = sometimes, 4 = often, 5 = very often. A mean of 4.62 signifies an average between often and very often. Satisfaction item scores were fairly high (mean range 4.06–4.62) and scores on burnout items were fairly low (mean range 1.73–2.44). However, a subgroup seems to be struggling. For example, 48% of respondents indicated feeling worn out and overwhelmed by work sometimes, often or very often (*n* = 112, 24, 9); 44% indicated feeling depressed because of the suffering of people they are trying to help sometimes, often or very often (*n* = 92, 29, 5); 24% indicated feeling trapped by their job as a helper sometimes, often or very often (*n* = 56, 13, 1).

#### Benefit finding/post-traumatic growth (PTG)

Respondents were asked about agreement with statements following a prompt, “as a result of the challenges in my life and/or job…” items included: handling difficulties, prioritizing what is important in life, appreciation for the value of own life, stronger than thought, learned how wonderful people are, and sense of closeness with others. Items were measured on a 6-point scale from “not at all” to “to a very great degree” 1 = not at all, 2 = to a very small degree, 3 = to a small degree, 4 = to a moderate degree, 5 = to a great degree, 6 = to a very great degree. Mean responses ranged from 3.8 to 4.35, indicating most respondents endorsed a moderate or larger amount of PTG and benefit finding in the midst of ongoing challenges. A typical distribution is captured in the item “I am stronger than I thought I was because of my experiences in life”: not at all 7% (*n* = 2), to a very small degree 2% (*n* = 6), to a very small degree 1% (*n* = 4), to a moderate degree 15% (*n* = 45), to a great degree 48% (*n* = 144), to a very great degree 33% (*n* = 99). Responses for burnout and PTG sections are summarized in Table 2.

**Table 2:** Burnout and satisfaction, benefit finding/post-traumatic growth.

**Item: burnout and satisfaction items (*N* = 300)**	**Mean (1–5 scale, 5 = very often)**
1. I get satisfaction from being able to help people	4.62
2. I believe I can make a difference through my work	4.30
3. I am able to balance my work and personal life	4.06
4. I feel worn out and overwhelmed by my work (45% sometimes/often)	2.44
5. I feel depressed because of the suffering of people I am trying to help (41% sometimes/often)	2.29
6. I feel trapped by my job as a helper (23% sometimes/often)	1.73
**Item: benefit finding/Post-traumatic growth items (*****N*** = **300)**	**Mean (0–5 scale, 5** = **to a very great degree)**
1. I know that I can handle difficulties	4.35
2. I prioritize what is important in life	4.27
3. I have an appreciation for the value of my own life	4.16
4. I'm stronger than I thought I was because of the experiences in my life	4.07
5. I have learned a great deal about how wonderful people are	4.07
6. I have a sense of closeness with others	3.83

### Organizational wellbeing: current resources and future initiatives

#### Perception of organizational support related to wellbeing

Survey participants shared extensive feedback for the organization related to wellbeing. When asked if the organization supports employee wellbeing 63% agreed. The remaining 37% percent breaks down as follows: 28% slightly agree, 5% neither agree nor disagree, 3% slightly disagree, and < 1% disagree. To explore perceived organizational support in greater detail, 16 questions were asked about wellbeing components using the following 5-point scale: 1 = disagree, 2 = slightly disagree, 3 = neither agree nor disagree, 4 = slightly agree, 5 = agree. The response range was high, from 4.27 to 4.65, indicating general agreement with all items such as “the organization supports employee wellbeing” (mean = 4.52). For additional details related to specific organizational components, see Table 3.

**Table 3:** Organizational support for wellbeing.

**Item: organizational support (*N* = 300)**	**Mean (1–5 scale, 5 = agree)**
1. The organization ensures that staff are clear about mechanisms for reporting any internal abuses	4.65
2. The organization provides regular supportive supervision	4.62
3. The organization ensures transparency of decision making in sharing with staff	4.60
4. The organization ensures job descriptions and reporting lines are clear and accurate	4.59
5. The organization provides fair and equitable salary scales	4.59
6. The organization promotes professional growth	4.54
7. The organization has provisions for grievance redress mechanisms or work-related conflicts	4.53
8. The organization supports employee wellbeing	4.52
9. The organization has provisions for material, financial and psychological support for staff	4.47
10. The organization creates opportunities for staff participation and feedback	4.43
11. There are programs in place through the organization to support employee wellbeing	4.41
12. There are policies in place through the organization to support employee wellbeing	4.39
13. The organization provides security/protection for staff	4.38
14. The organization creates opportunities for staff appreciation	4.33
15. The organization provides clear communication about working hours and leave time	4.33
16. The organization has some mechanism to support staff and their families if they are experiencing difficulties (such as a sick child)	4.27

#### Utilization of organizational resources for wellbeing

Of the total survey respondents, 19% have taken part in individual counseling sessions through the organization. When asked whether they agreed with the statement, “I found the individual counseling sessions helpful” 41% indicated somewhat, and 59% indicated very much. Additionally, 39% of staff have taken part in group supervision. In response to the statement, “I found the group supervision sessions helpful” 33% indicated somewhat and 66% very much.

#### Future wellbeing Intervention priorities

A list of 19 items was provided and survey respondents were asked if they were not at all interested = 0, somewhat interested = 1, or very interested = 2 in such content being delivered in a peer-faciliated group format framework. Mean responses were then compared for each item to come up with a rank order for the 19 items. Responses for an “other, specify” category (item 20) were also reviewed. Survey participants were also asked, “how interested are you (overall) in participating in such sessions?” Respondents indicated 3% not interested; 55% somewhat; and 42% very. See Table 4 for details.

**Table 4:** Future wellbeing intervention feedback (rank order preferences).

**Item: intervention content (*N* = 300)**	**Rank order preference, most preferred at top (mean, 2 = very interested)**
1. Learning about how to support children	1.69
2. Learning about sleep disturbance and how to support someone struggling	1.67
3. Opportunities to engage in fun social activities with colleagues	1.62
4. Learning new coping skills (e.g., how to feel calmer when under stress, such as breathing, grounding, mindfulness, muscle relaxation).	1.58
5. Psychological Preparedness among individuals, families, and teams. [“psychological preparedness” is defined as learning about and preparing to cope with psychological reactions to stressful situations]	1.55
6. Learning about facilitation of peer support intervention models for staff wellbeing	1.54
7. Learning about how humanitarian work and how personal exposure to war and displacement can affect wellbeing	1.52
8. Providing social support for the other staff within the organization and recognizing when a peer needs something more from an external mental health provider.	1.52
9. Learning about how to encourage healthy team dynamics and social cohesion and addressing within team conflict	1.51
10. Opportunity to connect with and exchange support with colleagues	1.50
11. Use of self and peer assessment tools for determining wellbeing and distress	1.49
12. Learning about loss and grief (including ambiguous loss)	1.47
13. Interactive content, involving games and activities	1.41
14. Opportunities to make suggestions to the organization's management about staff needs	1.40
15. Learning about and strengthening options for staff referrals to mental health professional for individual counseling (confidential; outside of the organization)	1.38
16. Learning about other topics relevant to wellbeing	1.37
17. Learning more about and/or receiving additional supervision related to your job, including any elements of staff wellbeing	1.34
18. Learning about and strengthening options for staff referrals to mental health professional for individual counseling (confidential but within the organization)	1.33
19. Learning about substance use and how to support someone struggling	1.23
20. Additional write in responses suggested by a few participants: learning about—*depression, suicide, loss, support for older persons, financial management, violence, family relationships, burnout*.	

### Using survey results to inform a peer support group wellbeing curriculum

Survey results informed the development of a *draft curriculum*, “Caring for the Carers: Peer Facilitated Group Intervention for Staff in Ukraine” (55). In addition to survey responses, the curriculum was based on a review of existing best practices, guidance, research, and related literature on mental health support needs for those working in conflict areas or similar settings. The content, organization of group sessions, mechanism of delivery and facilitation guidance were adapted to fit the specific needs identified in light of the situation in Ukraine (see Table 5 for an overview of curriculum content and objectives). Each peer support group session is designed to take place over a 2-hour in-person session facilitated weekly or biweekly. One hour of each session is dedicated to module content, with the first and last 30 min less formal, designed to enhance connection and socializing.

**Table 5:** Staff wellbeing curriculum: peer support groups.

**Session/Module**	**Objectives**
Overview Development of curriculum Structure of sessions Check-ins and current events Facilitator self-care Referral pathways	Global objectives for each session 1. Be open to learning about mental health topics through psychoeducation. 2. Reflect on the content, considering how the topic is personally relevant (self-awareness). 3. Be willing to practice new coping skills. 4. Make a commitment to prioritize mental health and wellbeing (at various levels: individual, family, workplace/team, and community).
Session/Module 1: understanding mental health and coping with stressors	1. Define mental health and wellbeing. 2. Identify common stressors and potential mental health impacts. 3. Understand risks to mental health associated with specific settings. 4. Consider common and preferred coping strategies.
Session/Module 2: psychological preparedness	1. Identify potential psychological and physiological reactions to extreme stress (immediate threat). 2. Identify forms of coping (grounding) that can override unhelpful responses to extreme stress. 3. Feel more confident in one's ability to be psychologically prepared to respond optimally in a crisis (optimal decision making under threat conditions).
Session/Module 3: part: social support and peer support skills	1. Learn how to provide and receive peer support in times of stress. 2. Identify and learn to utilize core peer support skills. 3. Understand the importance of maintaining confidentiality. 4. Understand when to refer out for additional support. 5. Consider ways to apply content knowledge to specific situations likely to be encountered during work.
Session/Module 4: Part II: social support and peer support skills	1. Learn how to identify, understand, and respond to signs of mental health distress and substance use challenges. 2. Learn when it is necessary to move beyond peer support and encourage engagement with professional service providers.
Session/Module 5: supporting systems: teams, families and communities	1. Understand how personal responses to stressors can disrupt team dynamics and undermine work. 2. Identify ways to implement peer support principles to foster cohesion and healthy communication in teams. 3. Identify how similar principles and additional techniques can be applied to families, clients, and community members.
Session/Module 6: bringing it all together: role plays and activities	1. Operationalize knowledge from previous modules.

### Curriculum development workshop

Noting the value of mixed methods, and the desire for data validation, the draft curriculum was workshopped with the Ukrainian team of healthcare and other humanitarian aid workers with the global health nonprofit supporting this work. In May 2023, four online training and consultation sessions were held with 16 Ukrainian staff selected as potential peer facilitators.

Most of these staff were psychologists, with some social workers and a few HR staff. Psychologist training in Ukraine typically involves a Master's degree in psychology, followed by specialized training in areas like clinical psychology. Several universities and organizations in Ukraine are actively involved in providing training and support to psychologists currently as the need for psychologists and related professions has increased in recent years. Additionally, the global nonprofit employer provides clinical supervision and other forms of support and ongoing training. This workshop participant group was selected as potential peer facilitators through a voluntary process.

During the 8 hour training (four sessions of 2 hours each), the focus was on: *presentation and interpretation of survey results (including reflecting on the validity and trustworthiness of the data), sharing of curriculum content and soliciting related feedback, associated discussions, and generating context specific case studies and other examples*. Many key changes to the draft curriculum were made and critical insights were gained during this direct engagement with staff. This step was essential in building on and expanding the survey results to tailor the curriculum to the specific needs of the staff and context.

A few examples are provided of how the workshop transcended a didactic training format, underscoring the value of participatory approaches to intervention (co)design. Example 1: during one workshop session, it became clear that staff were experiencing distress related to events occurring within Ukraine only a few hours prior. The first portion of the session was therefore spent encouraging staff to provide social support to one another, building on and highlighting interactions already occurring naturally within the group. This was used as an example of how the peer facilitators might approach the groups in a flexible manner, holding space for events happening in the lives of participants. This also helped to explain why having an informal start to the peer support groups (a 30 min “soft start”), can be beneficial.

Example 2: daily challenges staff participants shared during the cocreation workshop were woven into the updated version of the peer support curriculum. This provided a layer of nuance in excess of what was possible through the survey alone. Some of the qualitative data collected during the workshop that informed the peer support curriculum is summarized below.

#### Stress related to drone strikes

Workshop participants, potential peer facilitators, shared the chronic stress they struggled to cope with as a result of ongoing drone strikes. Some typical feedback is summarized in the following quotes: “huge distress from drone missiles, sometimes people don't or can't even go to the bomb shelters… it has been going on for so long and it just keeps going…;” “missiles at the time we are going to sleep, then quiet, then again in the middle of the night, we can't sleep, we are exhausted… my baby can't sleep, wakes up with sirens…;” with one person indicating, “evacuations are a huge source of stress…”

#### Inability to take time off

Those attending the workshop also shared that they felt unable to take time off of work, this representative quote illuminates the struggle: “It would be great to have a day off, a mental health day off… But there are huge tasks, and many others depend on me so I can't take a day off… we have no choice to not work, but a break would be nice…”

#### Media onslaught

Several times during the workshop participants emphasized stress associated with the constant media exposure to the conflict: “media exposure is causing so much distress… It is difficult to understand unless you feel it yourself. watching videos in the media, you feel as if you are there…;” “it is like somebody is torn away from your body when people die, it is not *just news*…” It was common for participants to share their concerns for colleagues, friends, family: “maybe you are in a safe place, no air sirens here, but colleagues are under shelling everyday…;” “when we call and ask how they are they say “we are good”—they are so brave.” “My son lives in Kiev, and I worry a lot.”

Participating staff also raised additional topics of interest during the workshop, including emphasizing the prevalence of alcohol and substance abuse and a desire for related curriculum content. Throughout the workshop participants weighed in to create case study examples to be used in the curriculum. They also helped in interpretation of survey data in specific ways. For example, some results seemed to suggest lower rates of distress than might be anticipated. Some of the Ukrainian staff consulted indicated that minimizing suffering is common in Ukraine, especially as many believe their concerns pale in comparison to the challenges facing those fighting on the front lines. A section addressing this was incorporated in the peer support curriculum. The survey and workshop resulted in an updated 6-session peer support group curriculum rolled out as a pilot initiative (detailed in Table 5) (55).

Throughout the workshop sessions, as indicated by examples above, feedback was collected on preferences and suggested improvements. There was a high degree of enthusiasm for interactive exercises, practical tools, group discussions, hearing ideas from colleagues, and the friendly and supportive atmosphere. Content was favorably rated across all modules, with particular enthusiasm for psychological preparedness and peer support skills. When asked about modifications, participants indicated a desire for a longer training, in-person components, and more interactive exercises. The Ukraine team has requested additional training for the peer support model beyond the 6 modules. The peer support curriculum is part of the broader C4C initiative which includes rest and recuperate policies, individual counseling/supervision and flexible working hours.

In terms of additional work to date and next steps, additional training sessions and have been provided to staff in Ukraine and the peer support intervention is in the process of being adapted and rolled out in two other countries. The organization plans to conduct a thorough review of outcomes and impact of the peer support intervention in the near future.

## Discussion

The aim of this work was to solicit feedback from healthcare and humanitarian aid workers in Ukraine to codesign a peer support curriculum. The majority of Ukrainian staff highlighted in this case study were engaged in community-based in-person work, including significant numbers of psychologists and social workers providing mental health, psychosocial, gender-based violence and child protection programming through mobile clinics. Survey and participatory workshop results indicate such staff are facing stressors similar to those reported by other healthcare and humanitarian aid workers, although security and other conflict specific concerns appear to be especially pronounced for this sample (11, 13–15). For example, top stressors included: *personal safety and security* (43%), *concerns for family and friends* (32%), and *financial hardships* (29%). Just over one-third of respondents indicated that stress was interfering with their ability to do their job. Common forms of coping included *distraction* (73%), and *use of alcohol or drugs* (32%). Nearly all (97%) indicated interest in a peer support group intervention. In addition to interest in socializing with colleagues, top preferences for curriculum content included: *coping skills, psychological preparedness, peer support facilitation skills, and healthy team dynamics*.

Results on the impact of stress, including burnout, mirror findings from research with workers in similar settings, underscoring the heightened risk of adverse mental health outcomes among this group (12, 16). However, survey results are variable, with some responses suggesting lower rates of distress, and greater satisfaction, benefit finding and post-traumatic growth than might be expected within this population during a period of escalating violence in the Russo-Ukrainian War.

Survey results were used to develop a 6-module peer group curriculum, refined through subsequent Train the Trainers workshops. Participatory approaches, such as the curriculum development workshop, provided an opportunity to gather additional qualitative information and interpret survey results. This process provided useful insights, including feedback indicating that cultural and contextual factors may point to a tendency to minimize distress.

This work is aligned with recent initiatives in Ukraine promoting mental health and wellbeing country-wide. For example, in 2017 Ukraine adopted a Concept Note on Mental Health Care in Ukraine up to 2030; shortly thereafter the related Action Plan for 2021–2023 and its extension for 2024–2026 was adopted to support the Concept Note. In 2025, a new law on the Mental Health Care System in Ukraine passed. The Universal Mental Health Training (UMHT) was subsequently rolled out, an educational program similar to mental health first aid, for frontline professionals including emergency responders, social workers, and others (56). This peer support staff wellbeing curriculum approach can complement and otherwise support such efforts nationwide, tailored to distinct groups of workers.

This case study provides information on a peer support intervention development process. However, limitations exist, including several flagged here. For example, survey questions were refined/adapted with Ukrainians but there was no formal process for norming and validation of survey measures for the Ukrainian context. Additionally, this manuscript does not include a description of the full implementation roll out phase or any program evaluation elements. Such information will be included in subsequent publications.

## Conclusion

This survey and follow on workshop approach underscores the value of using mixed methods to cocreate interventions tailored to specific groups. This case study provides a unique example of the development of a participatory, peer-led intervention to support healthcare and humanitarian aid workers in conflict zones—a model that can be adapted for use across contexts and with a variety of workers.

## Data Availability

The raw deidentified data supporting the conclusions of this article will be made available by the authors, without undue reservation.
